# Gender equity and sexual and reproductive health in Eastern and Southern Africa: a critical overview of the literature

**DOI:** 10.3402/gha.v7.23717

**Published:** 2014-06-26

**Authors:** Eleanor E. MacPherson, Esther Richards, Ireen Namakhoma, Sally Theobald

**Affiliations:** 1 Department of International Public Health, Liverpool School of Tropical Medicine, Liverpool United Kingdom; 2 Research for Equity and Community Health Trust, Lilongwe, Malawi

**Keywords:** gender equity, sexual & reproductive health, Eastern and Southern Africa

## Abstract

**Background:**

Gender inequalities are important social determinants of health. We set out to critically review the literature relating to gender equity and sexual and reproductive health (SRH) in Eastern and Southern Africa with the aim of identifying priorities for action.

**Design:**

During November 2011, we identified studies relating to SRH and gender equity through a comprehensive literature search.

**Results:**

We found gender inequalities to be common across a range of health issues relating to SRH with women being particularly disadvantaged. Social and biological determinants combined to increase women's vulnerability to maternal mortality, HIV, and gender-based violence. Health systems significantly disadvantaged women in terms of access to care. Men fared worse in relation to HIV testing and care with social norms leading to men presenting later for treatment.

**Conclusions:**

Gender inequity in SRH requires multiple complementary approaches to address the structural drivers of unequal health outcomes. These could include interventions that alter the structural environment in which ill-health is created. Interventions are required both within and beyond the health system.

Eastern and Southern Africa (ESA) contains some of the poorest countries in the world. The burden of disease in these settings due to infectious diseases and diseases related to reproductive and sexual health is extremely high. Notably, all 10 countries with the highest prevalence of HIV are found in the region, and the high rates of population mortality related to the epidemic have had a devastating impact on socio-economic development. Further, as the region with the highest number of young people in the world, understanding the drivers of sexual and reproductive health (SRH), including gender inequality, could provide valuable insights for improving broader health and development outcomes.

This review of the literature draws on findings from a report commissioned by the Regional Network for Equity in Health in Eastern and Southern Africa (EQUINET) which highlighted areas of concern for gender equity in health in ESA, based on a review of the published and grey literature (1). The paper synthesises evidence from the academic and grey literature on key aspects of SRH from 16 countries in the Eastern and Southern African region and highlights neglected areas where further progress is urgently required.

Gender refers to how a person's biological sex is culturally valued and interpreted into locally accepted ideas of what it is to be female or male (2). Gender therefore describes all the socially given attributes, roles, activities, and responsibilities connected to being male or female in any given society (3). In most societies, these gendered social norms divest greater privileges (often power and resources) to men and boys over women and girls. Gendered social norms are dynamic, changing over time, and varying across cultures (2, 4). Gender differences in access to information and resources (both social and financial) can impact on nutrition, education, employment, and income. These are all important determinants of good health (5). There is therefore a need to better understand how gender shapes vulnerability to ill-health and health sector responses so that health services can address the needs of women, men, girls, and boys more equitably (i.e. through channelling resources where they are most needed). Gender equity denotes equivalence in life outcomes for women and men, recognising their different needs and interests, and requiring a redistribution of power and resources (2).

SRH relates to the health and well-being of people in matters related to sexual relations, pregnancy, and birth. The ability of women to realise their sexual and reproductive rights is vital to achieving gender equity in health as well as the empowerment of women. The aim of this paper is to critically review the published and grey literature on gender and SRH in ESA, identifying key areas of concern, areas of innovation and action, and charting the way forward. We discuss using a gender lens the following key components of SRH: access to abortion, maternal mortality, unmet contraceptive needs, gender-based violence, and HIV (including prevention, access to anti-retroviral therapy (ART), and caring responsibilities).

## Methods

During November 2011, we conducted a literature search of electronic databases for academic literature on gender equity and health in ESA. We developed key search terms which included sex, gender, men, women, reproductive health, disease, Southern and Eastern Africa. The two databases we used for these searches were Medline and Web of Knowledge. We also used a Google Search to ensure we included a range of literature.

We limited the searches to include the following 16 countries in ESA: Uganda, the Democratic Republic of Congo (DRC), Kenya, Tanzania, Zambia, Malawi, Mozambique, Madagascar, Mauritius, Angola, Namibia, Botswana, Swaziland, Lesotho, South Africa, and Zimbabwe. We focused on these countries because they provided a range of countries including those with more and less stable political and economic systems and higher and lower HIV epidemics. We included studies for the review that related to gender equity and health (both inside and beyond the health system) in the 16 countries and excluded those that did not relate to gender equity and health.

Once we had identified key papers, we reviewed each included paper through undertaking a thematic analysis. As a group, we discussed the key findings and identified broad themes to include in the final write up.

In this paper, we have focused specifically on our findings related to SRH. We identified key themes in this area including: measures of gender inequalities; maternal mortality; abortion; unmet contraceptive needs; HIV risk, treatment and care; and gender-based violence. We brought these findings together and discussed as a group to identify key priorities for action to improve the response to address gender inequalities in SRH. Key limitations of this review were: first, the lack of primary data available for the ESA settings related to gender and health, although there were more data available for South Africa and Kenya than for other countries; second, there was a lack of empirical work that identified positive gender equity action in health; thus, the review was largely reliant on descriptive case studies; third, while men and boys are also affected by gender power relations, there were few studies available that focused on men and how gendered norms and expectations can impact on their health; fourth, in reviewing the papers, there were limited papers relating to sexually transmitted diseases other than HIV/AIDS and hence the focus is on HIV rather than the broader set of sexually transmitted infections (STIs).

## Results

Using the search strategies, we identified 1,291 potentially relevant published articles. All titles and abstracts were read and any duplicates or papers that were not relevant to gender and health were omitted. This left 189 unique papers that were reviewed in full before removing a further 40 manuscripts with irrelevant or repeated data. We also identified 26 papers from the grey literature by conducting Google searches and reviewing references in journal articles. The results presented are key themes related specifically to SRH.

### Introducing the ESA countries through the Gender Inequality Index

The Gender Inequality Index (GII) is part of the United Nations Development Programme (UNDP) and is constructed based on three dimensions – reproductive health (maternal mortality and adolescent fertility), empowerment (female parliamentary participation and secondary level education), and women's participation in labour markets (6). A higher score on the index means that gender inequality is more pronounced in the country. While the index includes only a limited number of indicators of gender inequality, it provides a useful summary of gender inequities across countries. It indicates that in countries where human development is uneven there is also high inequality between men and women. Table 1 below presents UNDP GII for the 16 countries. Mauritius has the lowest gender inequality and the DRC has the highest. The civil war in DRC is likely to play an important role in explaining why the country performs poorly in this index. War can be especially damaging to the rights of women and children. In DRC, there have also been extensive reports of rape, sexual slavery, purposeful mutilation of women's genitalia, and killings of rape victims, particularly in eastern Congo (7). In comparison, Mauritius has a stable democracy with one of the highest per capita incomes in Africa.

**Table 1:** UNDP Gender Inequality Indices, 2011

EQUINET focal countries	UNDP Gender Inequality Index 2011
Democratic Republic of the Congo	0.71
Kenya	0.63
Zambia	0.63
Mozambique	0.60
Malawi	0.59
Tanzania	0.59
Uganda	0.58
Zimbabwe	0.58
Swaziland	0.55
Lesotho	0.53
Botswana	0.51
South Africa	0.49
Namibia	0.47
Mauritius	0.35
Angola	Data unavailable
Madagascar	Data unavailable

Source: UNDP (6).

### Maternal mortality

Women's access to antenatal services and support during labour are vital elements of SRH rights. Maternal mortality is defined as the death of a woman during pregnancy, childbirth, or in the first 42 days after giving birth (8). The maternal mortality ratio (MMR) is the number of maternal deaths in a population divided by the number of live births (9). It is one of the health indicators that shows the greatest gap between the rich and the poor – both between countries and within them (10). This is highlighted by the fact that 99% of all maternal deaths occur in developing countries, with more than half of these deaths occurring in sub-Saharan Africa (9). A woman's lifetime risk of maternal death is 1 in 7,300 in developed countries versus 1 in 75 in developing countries (10). Power dynamics within the household can mean that women are unable to take control over their reproductive health, putting them at increased risk of maternal death.

With the adoption of Millennium Development Goal 5 (MDG 5), countries have committed to reducing the MMR by three quarters between 1990 and 2015 (11). However, between 1990 and 2005, the MMR declined by only 5%. Achieving MDG 5 requires accelerating progress. With the exception of Mauritius, none of the ESA countries in this review are likely to achieve their MDG5 target (8).

In Table 2, the MMR of the 16 countries in the study are represented for 1980, 1990, 2000, and 2008. There has been a downward trend in mortality in some of these countries, including Madagascar, Angola, Mauritius, Uganda, Kenya, and Tanzania. Although in relation to Kenya the gains made are slight, and between 1990 and 2000 there was a significant increase in maternal mortality. Some of the improvements in maternal mortality in these countries may be explained by a number of factors. Hogan et al. (8) argue that globally total fertility rates have been declining since 1980 (3.70 in 1980 to 3.26 in 1990 and 2.56 in 2008). There has also been an increase of per capita income (although this is less marked in ESA countries than in other regions of the world). Rising per capita income can impact on maternal mortality through improving women's nutritional status and their financial access to health. Educational attainment may also play a role in these improvements (maternal educational attainment is a strong correlate of maternal mortality) and has been rising in sub-Saharan Africa since 1980 (12).

**Table 2:** MMR of the 16 countries in 1980, 1990, 2000, and 2008

	MMR per 100,000 live births			
		
Country	1980	1990	2000	2008
Angola	1,309	1,156	1,105	593
Botswana	424	237	655	519
DRC	498	616	850	534
Kenya	494	452	730	413
Lesotho	588	363	1,021	964
Madagascar	490	484	505	373
Mauritius	122	65	34	28
Malawi	632	743	1,162	1,140
Mozambique	411	385	505	599
Namibia	397	354	558	586
South Africa	208	121	155	237
Swaziland	559	359	609	736
Tanzania (United Republic of)	603	610	714	449
Uganda	435	571	604	352
Zambia	599	594	914	603
Zimbabwe	219	171	373	381

Source: Hogan et al. (8).

MMR, maternal mortality ratio.

The countries with upward trends of worsening maternal mortality include Botswana, DRC, Lesotho, Malawi, Mozambique, Namibia, South Africa, Swaziland, Zambia, and Zimbabwe. With the exception of DRC, all of these countries lie in Southern Africa and have some of the highest HIV prevalence rates in the world. The high HIV prevalence is likely to be a significant factor in shaping these poor outcomes in maternal mortality (12). Therefore, interventions to prevent HIV transmission and the early initiation of ART are likely to be important interventions to reduce maternal mortality. While HIV provides some explanation for persistent high levels of maternal mortality in the majority of ESA countries, it is clear that other factors contribute to this, which we discuss below.

Nearly half of all maternal deaths in developing countries, including the ESA region, occur during labour, delivery, or the immediate post-partum period (13). The ‘three delays’ model developed by Nour^1^ (14) reflects the important role that gender relations play within the household (14). Women face significant challenges in accessing health services at these critical stages which is shaped by their access and control of resources, as well as the prevailing gender norms and values. For example, economic resources and their distribution within the family may prevent or delay women from seeking treatment (14). Furthermore, cultural and religious norms may influence the availability and accessibility of key health interventions such as contraception as well as abortion procedures.

### Abortion

In 2003, there were an estimated 20 million unsafe abortions. Nearly 98% of them were in the Global South in countries which have restrictive abortion laws (15). Nearly 200 women die each day from abortion-related complications (16). Africa accounts for 25% of all illegal abortions performed worldwide and less than 1% of all legal abortions (17). An estimated 90% of deaths from unsafe abortions and 20% of obstetric mortality could be averted by universal access to modern family planning methods. As can be seen in Table 3 on abortion laws in the 16 ESA countries, every country allows abortion on the grounds of saving a woman's life (18). However, there is wide variation in whether women can get an abortion for any other reason. Angola, DRC, and Lesotho have the most restricted while South Africa has the most liberal abortion laws. In 1996, following the end of apartheid rule and the transition to democracy, the South African government introduced the Choice on Termination Act, No. 92, 1996, which granted abortions on a number of grounds, including on request.

**Table 3:** Legal grounds for abortion, 2007

	Grounds on which abortion may be permitted in 16 review countries						
	
Countries	To save a woman's life	To preserve physical health	To preserve mental health	Rape or incest	Foetal impairment	Economic or social reason	On request
Angola^a^	√	×	×	×	×	×	×
Botswana	√	√	√	√	√	√	×
Democratic Republic of Congo^a^	√	×	×	×	×	×	×
Kenya^b^	√	√	√	×	×	×	×
Lesotho	√	×	×	×	×	×	×
Madagascar^a^	√	×	×	×	×	×	×
Malawi^a^	√	×	×	×	×	×	×
Mauritius^a^	√	×	×	×	×	×	×
Mozambique	√	√	√	×	×	×	×
Namibia	√	√	√	√	×	×	×
South Africa	√	√	√	√	√	√	√
Swaziland	√	√	√	×	√	×	×
Tanzania	√	√	√	×	×	×	×
Uganda^b^	√	√	√	×	×	×	×
Zambia	√	√	√	×	√	√	×
Zimbabwe	√	√	×	√	√	×	×

Source: WHO (15).

a The abortion laws in these countries do not expressly allow abortions to be performed to save the life of the woman, but general principles of criminal legislation allow abortions to be performed for this reason on the grounds of necessity.

b The abortion laws in these countries expressly allow abortions to be performed only to save the life of the woman, or are governed by general principles of criminal legalisation which allow abortions to be performed for this reason on the ground of necessity.

In South Africa, clause 59.1 of the post-apartheid Constitution (1996) requires that Parliament facilitate public involvement in legislative and other processes of the assembly and its committees. Women's rights groups successfully used this clause to mobilise for legalisation of abortion. One of the key strategies was to allow women who have experienced unsafe abortions first hand to speak about the need for liberal abortion laws before Parliament. These women also provided quantitative evidence of costs to the government (that could be avoided) due to complications of unsafe abortion. As a result, South Africa thoroughly liberalised its abortion laws. The Choice on Termination Act No. 92, 1996, allows abortion on request up to the first trimester, permits midwives to conduct abortions, and allows adolescent girls the right to access abortion without parental consent.

Jewkes and Rees report a 91% reduction in deaths from unsafe abortion from 1994 (before the Termination Act had been passed) to 2000 (once the Act was in operation) (19). They compared abortion-related deaths from more than one source (Department of Health reports on confidential enquiries into maternal deaths from 1999 and 2003 and the national incomplete-abortion survey from 2000), with the estimates of pre-legislative reform mortality found in the national incomplete-abortion survey from 1994. The 1994 survey estimated that there were 425 deaths each year in public facilities from unsafe abortion. When the survey was repeated in 2000, no deaths were detected in the 3-week data collection period in any study hospital. Therefore, researchers were able to conclude that a significant decline in mortality had occurred although it was not possible to estimate the annual number of deaths (19, 20).

### Unmet contraceptive needs

**Fig. 1:**
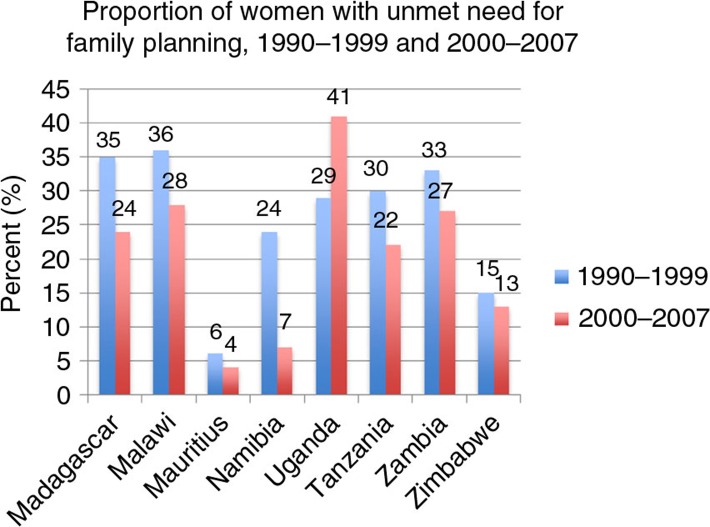
Proportion of women with unmet family planning needs, 1990–1999 and 2000–2007. Source: WHO (22).

Unmet contraceptive needs can be used as a proxy measure for accessing family planning services (21). This is because most unplanned pregnancies in the developing world are due to lack of access to family planning services. As shown in Fig. 1, where data are available, there is a gap in unmet family planning needs (22).

This is particularly pronounced in Uganda where unmet need has increased from 29% in 1990–1999 to 41% in 2000–2007. The studies we identified on this topic did not explicitly explore the reasons for this increase. However, women who desire to space or limit births may face multiple barriers both within their relationships and also related to the wider provision of health services they have access to. Meeting women's family planning needs is vital for empowering women. In countries where abortion is restricted, as shown in Table 3, unmet contraceptive need can mean women are more likely to have unsafe abortions. It can also mean that women are at an increased risk of maternal mortality.

It is critical that SRH services are accessible for all. In ESA countries this means improving the coverage of family planning services to widen their availability, particularly for hard-to-reach populations, as well as improving their accessibility and responsiveness to the needs of users. Ostlin (10) argues that in some countries the introduction of women and adolescent friendly services has helped to counteract judgmental attitudes of providers, and lack of privacy and confidentiality (10). Services could be adapted to include youth-only, men-only clinics or women-only services or outreach and community-based services (10).

### HIV risk, care, and prevention

Globally, the response to HIV and AIDS has triggered an unprecedented focus on gender inequality and how this shapes women's vulnerability (23). This response has not been mirrored in other aspects of disease prevention or within health systems more generally. One of the reasons for this unparalleled response is the statistics relating to HIV. Of the estimated 34 million people living with HIV (PLWHIV) worldwide (24), women constitute half of all adults living with the disease. However, in sub-Saharan Africa, there are 14 HIV-positive women for every 10 HIV-positive men (25). Seventy-five per cent of new HIV infections occur among young girls and female adolescents in Southern Africa (25). Women aged 15–24 are twice as likely to be infected with HIV than boys of the same age in the region (25). The higher risk in young women is due to both sociological and physiological risk factors – young women have immature genital tracts but are more likely to have older sexual partners partly due to gendered expectations of men's and women's roles as men are expected to have younger sexual partners (26).

As can be seen from Table 4, HIV prevalence in the 16 Southern and Eastern African countries varies widely from 0.2% for Madagascar and 1% for Mauritius to 25.9% for Swaziland and 23.6% for Lesotho (24). Southern Africa has borne the brunt of the HIV epidemic globally. Likewise, ART coverage varies significantly, with Botswana (93%) and Namibia (90%) performing best, and Madagascar (1%) the worst. South Africa scored a disappointing 55%, despite having the third highest HIV prevalence in the region (17.8%).

**Table 4:** Prevalence of HIV and percentage of ART coverage, 2010

Country	Prevalence (%)	ART coverage (%)
Angola	2	33
Botswana	24.8	93
DRC	No data available	No data available
Kenya	6.3	61
Lesotho	23.6	57
Madagascar	0.2	1
Malawi	11	No data available
Mauritius	1	16
Mozambique	11.5	40
Namibia	13.1	90
South Africa	17.8	55
Swaziland	25.9	72
Tanzania	5.6	42
Uganda	6.5	47
Zambia	13.5	72
Zimbabwe	14.3	59

Source: UNAIDS (24).

Gendered roles and relations often place women in a subordinate position to men and promote models of masculinity that justify and reproduce men's dominance over women (27). As Grieg et al. argue, ‘notions of masculinity prevalent in many parts of the world that equate being a man with dominance over women, sexual conquest and risk-taking are associated with less condom use, more STIs, more partners, including more casual partners, more frequent sex, more abuse of alcohol and more transactional sex’ (27). Women are often economically dependent on men. This dependence can leave women with less power in sexual relationships, particularly in negotiating the terms of sexual exchange including condom use (28). In many ESA countries, masculine and feminine ‘ideals’ reflect views that promote the sexual prowess of men through multiple sexual partnerships, but frown on women who do the same (29).

Recent work has identified violence against women as an independent risk factor for HIV in South Africa (30, 31). Within a sexual relationship, the threat of violence can influence women's power and their ability to negotiate conditions of sexual intercourse, especially condom use (32). Women who face violence within their relationships may be less likely to access HIV testing services because of the fear of disclosing their sero-status (33). Dunkle and Jewkes present research from India, South Africa, and the United States suggesting that ‘men who are violent towards their female partners or commit rape’ are more likely to have sex more often, to have sex with concurrent and/or casual sexual partners, to have higher total numbers of sexual partners, to practice anal sex, to participate in transactional sex, to father children, and to use alcohol and drugs’ (33). These are all factors that increase risk of HIV.

Prevention of HIV has relied largely on behaviour change at the individual level. However, behaviour change interventions have often been gender-blind and have failed to acknowledge the broader environment that sexual relationships are conducted within. Gender power relations and economic vulnerability mean that women are often unable to change their behaviour particularly relating to condom use. A study in a peri-urban area in Malawi found that women can do very little to influence condom use by their husbands to protect themselves from HIV due to the perception that condom use implies infidelity; nor can they space or stop having children without their male partner's permission (34). Studies in Kenya and South Africa have found that men control condom use in sexual exchanges (35, 36).

Nevertheless, there are examples from South Africa of promising HIV prevention interventions with both women and men. For example, Stepping Stones is a socially transformative programme that promotes gender equality through participatory learning and action (37). The intervention works with community members, including men and boys. In South Africa a Stepping Stones intervention was evaluated with a group of rural youth using a randomised controlled trial. The trial found that with 2 years follow-up, Stepping Stones lowered the incidence of herpes simplex virus 2 in men and women by approximately 33%, and men reported less perpetration of intimate partner violence across 2 years of follow-up, as well as changes in several other HIV risk behaviours (37).

The gendered differences in financial inequality, authority relations, and social identities of men and women influence how families, communities, and health care systems react towards HIV infection among men and women (38). Views of blame and accusation have in some societies been directed more towards female PLWHIV than male PLWHIV (38).

However, gender roles and relations also influence access to HIV testing, treatment, and care. Studies have shown that more women receive treatment than men in ESA, and that when men do access treatment they often do so at a more advanced stage (39). This may reflect dominant masculine norms that prescribe not only an avoidance of the sick role but also discourage men from seeking care out of fear of being labelled weak.

Finally, care for HIV in ESA has gradually been shifting away from providing hospital-based care to home-based care (40). While this model of care has clear benefits for patients who wish to remain at home, it has often relied on women's unpaid labour reinforcing the stereotype that women are responsible for caring for the sick and that work of this nature is not worthy of remuneration (41–43).

### Gender-based violence

While gender-based violence is pervasive in many ESA countries, it is often under-recognised as a public health challenge. The extent to which women are exposed to violence varies across countries. The data indicate, however, that violence against women is widespread and manifests in many forms – physical, sexual, psychological, and economic – both within and outside the home. Violence limits women's autonomy and their ability to make decisions about their bodies. It can also have wide ranging impacts on the short and long term physical, mental, and sexual health problems of women (44).

These health problems can range from physical injuries to depression and suicide. Living with the threat of violence can also leave women unable to negotiate the terms of sexual relationships as well as contraceptive use. This can leave women vulnerable to unwanted pregnancies as well as sexually transmitted diseases (45).

According to Duggan (45), perpetrators of violence against women are most often their intimate partners; therefore, violence may be considered one of the ‘most graphic expressions of unequal household power relations’ (45). Women are abused physically and sexually by intimate partners at different rates throughout the world – yet such abuse occurs in all countries or areas, without exception. There are limited data available on the occurrence of violence against women in sub-Saharan Africa, but the United Nations Children's Fund estimates 13–49% of women reported having been physically assaulted by an intimate male partner (46). Studies on sexual violence in Ethiopia, Kenya, Namibia, Tanzania, Zambia, and Zimbabwe estimate that 14–59% of women have experienced sexual violence at some point during their lives (46). Pregnancy can also be a trigger for violence with 10% of ever-pregnant women in Zimbabwe and 7% in South Africa having been attacked during pregnancy (46).

South Africa has one of the highest rates of rape in the world and in a recent study 27.6% of men interviewed admitted raping a woman (47). This involved an intimate partner, stranger, or acquaintance, and the rape was perpetrated either alone or with accomplices. Further, 4.7% men admitted raping a woman in the past 12 months. In DRC, there have been reports of rebels using rape as a weapon of war to humiliate women and girls as well as to humiliate the women's spouses. It has also been used as a tool to terrorise and demoralise whole communities (48).

Action on gender-based violence is required at all levels, including community, legal, and health system levels. There are examples of such action in ESA countries. For example, an assessment in Kenya in 2003 revealed limited post-rape services, lack of policy, and tensions between HIV and reproductive health staff at service delivery points. Facilities lacked protocols and confidential spaces for treatment. In response, a standard package of care was developed with Liverpool Volunteer Counselling and Testing (LVCT). This included the provision of HIV post-exposure prophylaxis (PEP), psycho-social support, and gender-sensitive counselling for survivors (49). By June 2007, there were 13 health facilities providing post-rape care services in Kenya including the national referral and teaching hospital. Between them they had delivered services to over 2,000 adults and children, with 96% of those eligible initiating PEP at presentation (49).

## Conclusions

Synthesising published and grey literature from across the 16 ESA countries reveals that biologically and socially determined gender roles and relations influence the different exposures and vulnerabilities to illness experienced by women and men. Access to SRH is important for women and men of different ages. In ESA where maternal mortality rates and HIV prevalence remains unacceptably high, there is an urgent need to provide effective and accessible maternity services and gender-transformative HIV programmes. Gender norms and values influence availability and accessibility of critical SRH interventions such as contraception and abortion procedures. Within the context of the ESA countries, gender roles and relations play an important role in producing vulnerability to unwanted pregnancy and STIs. Notions of masculinity often condone multiple sexual partnerships and unsafe sexual practices, such as sex without a condom. They also influence treatment-seeking behaviour with men presenting later for testing and treatment, which in turn can influence treatment outcomes. Gender roles, which influence women's access to economic resources, can also increase vulnerability to HIV and other STIs because women may be more economically dependent on men and therefore less able to negotiate the terms of sexual exchanges.

Globally, the politics of SRH have always been contentious, and within the ESA contexts this is illustrated, for example, by the majority of countries limiting women's safe access to abortion. Of all the public health interventions, those related to SRH are the most likely to be influenced by politics, tradition, and religion rather than scientific evidence, especially where gendered issues are concerned. The unease that many people feel about discussing sex and the low status of women in many countries has meant that SRH rights have not always been enacted (50–52).

Gender inequity in SRH in ESA underlines the need for deeper levels of action to address the structural drivers of inequities in health outcomes. These could include interventions to transform gender roles and relations. Programmes such as Stepping Stones have good potential here through changing broader gender norms. Other interventions to address social determinants include improved access to education and employment.

It is clear from these ESA findings that interventions to improve SRH for women and men need to be enacted both within and beyond the health system. Tolhurst et al. (53) provide pertinent insights on the importance of advocacy and inter-sectoral approaches (53). They argue for increased advocacy in policy-making, service provision and resource allocation to establish the sexual and reproductive rights of women, men, boys and girls and to ensure these are met (53). Intervening requires moving beyond simple service delivery through using inter-sectoral collaboration to improve women's bargaining power and increase their broader access to and control over resources (53).

The causes of gender-based violence point to the need for multi-pronged approaches, including action at the community level to change cultural norms, decrease the acceptability of violence against women and girls, and improve the linkages between the health system and the legal system. Changing such norms calls for political, civil, and social leadership to change institutional practice and to debunk underlying ideologies. A common indicator of improved governance is the increase in women's participation at all levels of decision-making, including at the household and community levels and in national legislative bodies. However, wider transformations are needed to strengthen institutional functioning to address gender equity. Such transformations rely on the development of institutional capacity and governance based on robust evidence and examples of effective good practice. This includes advocacy for health information systems disaggregated by gender and by other important factors that shape vulnerability and resilience to ill-health (poverty, age, literacy, disability).

In conclusion, SRH is central to gender equity in health in the ESA region. Women's and men's access to these services remains sub-optimal which has serious health consequences for all, but particularly for women and girls. Access to these services is particularly important in ESA countries where HIV prevalence remains unremittingly high. It is vital to audit gender equality within the health system, to encourage programmes to carry out such assessments, to implement gendered evaluations, and to consult and obtain feedback from women and men in the community to strengthen gender equity in sexual and reproductive health.

## Footnotes

1 Nour (14) argues that there are three key delays that increase maternal mortality in resource-poor settings. These are: delays in deciding to seek care; delays in reaching care in time; delays in receiving adequate treatment.
